# Influencing Mechanism of Justice Sensitivity on Knowledge Hiding in the Chinese Context

**DOI:** 10.3389/fpsyg.2021.802171

**Published:** 2022-02-03

**Authors:** Zhang Jin-song, Huang Hua, Ruan Dan-yang, Jin Ya-nan

**Affiliations:** ^1^School of Management, South-Central University for Nationalities, Wuhan, China; ^2^School of Business Administration, Zhongnan University of Economics and Law, Wuhan, China; ^3^Clean Production Research Center in Wuhan Optical Valley and the Soft Science Research Base of Low-Carbon Development and Cleaner Production, Wuhan, China

**Keywords:** perpetrator sensitivity, victim sensitivity, territoriality, perceived time pressure, knowledge hiding

## Abstract

Good knowledge management is important for enterprises to maintain competitive advantage; however, the knowledge hiding behavior may hinder this process. Based on the conservation of resources and psychological ownership theories, using a chain intermediary model, this study investigates the effect of justice sensitivity on knowledge hiding through perceived time pressure and territoriality, and further tests the moderating role of territoriality. For the study, we collected 436 questionnaires from China through the Wenjuanxing Sample Service, of which 391 were valid. We then conducted multiple regression analysis and employed the bootstrap method for our tests. The results show that victim sensitivity has a significant effect on perceived time pressure, territoriality, and knowledge hiding, and that a chain mediating effect of perceived time pressure and territoriality is established between justice sensitivity and knowledge hiding. Further, territoriality has a positive moderating effect on perceived time pressure and knowledge hiding, while the mediating effect of perceived time pressure on justice sensitivity and knowledge hiding is also moderated by territoriality. Further, the study offers important practical implications in that enterprises should not blindly pursue results by making employees work excessively overtime. And there should have rationalized regulations in organization to ensure justice. The management should pay close attention to the psychological problems of victim and perpetrator. Instead, enterprises should have a certain degree of control, offer rationales for overtime work, and give high wages to the employees to compensate for their time, thus making the employees feel the worthiness of their overtime work and reducing the probability of engaging in knowledge hiding behaviors.

## Introduction

With the rapid development of the Internet economy in recent years, knowledge management methods have become increasingly important for enterprises, governments, and non-governmental organizations to remain competitive. In particular, efficient knowledge transfer and sharing among members of an organization can improve performance level and innovation capabilities (Ke et al., 2007; Wang and Yan, 2020). One way for enterprises to achieve sustainable development is to create knowledge through extensive knowledge sharing and exchange among employees, thus organizing knowledge to add greater value. However, knowledge hiding hinders the effectiveness of organizational knowledge management. Therefore, enterprises not only need to promote knowledge sharing, but also need to reduce and control employees’ knowledge-hiding behavior (Fu et al., 2020) which can affect the sustainable knowledge sharing within a research team by reducing the supply of knowledge, creating a poor knowledge-sharing atmosphere and forming an interpersonal distrust relationship (Liu et al., 2020).

On the one hand, scholars began to explore the reasons for employees to hide their knowledge and the construction of this concept. Connelly et al. (2012) defined the concept of knowledge hiding as an individual’s deliberate hiding or hiding of the knowledge asked by others, and developed a scale of knowledge hiding. The factors influencing knowledge hiding can be divided into the following aspects: factors related to knowledge, interpersonal relationship factors, situational factors, and personality characteristics. Current research mostly focuses on interpersonal and situational factors; however, the structure of factors influencing knowledge hiding behavior has not been fully studied despite its significance, as such behavior is ubiquitous in organizations and may affect results at the individual and organizational levels (Kumar Jha and Varkkey, 2018). An example of a counterproductive behavior of knowledge workers is the unwillingness to share knowledge with others or give false information, which overlaps with the concept of knowledge hiding (Peng, 2011). However, knowledge hiding is not always negative, as the motives may include prosocial ones(Peng, 2013), thus they can be both positive and negative. In the study of counterproductive behaviors, they are often combined with organizational justice (Liu et al., 2011). Organizational Justice has a direct negative impact on Knowledge Hiding (Oubrich et al., 2021). Justice sensitivity, which is defined as people’s sensitivity to unfair events, is related to both prosocial and antisocial behaviors, with previous studies showing that fairness is a stable personal trait. Nevertheless, despite the importance of evaluating the influence of justice sensitivity on knowledge hiding, it has not been sufficiently explored in the existing literature.

On the other hand, the theory of resource conservation covers a wide range of fields. Hobfoll (1989) proposed the theory of resource conservation to explain pressure and how individuals respond accordingly when facing it. According to Hobfoll (1989), employees perceive pressure in the following four situations: ① when individuals perceive the threat of resource loss; ② when resources are lost; ③ when individuals perceive that they need to invest more resources in the work; ④ when an individual’s perceived input is inconsistent with output. In addition, justice sensitivity occurs when people experience reaction intensity due to unfair events, which can be defined as people gain or lose resources, with many studies verifying the role of knowledge hiding as indicated by the conservation of resources theory (Škerlavaj et al., 2018; He and Gao, 2019).

In addition, De Clercq et al. (2019) investigated the relationship between time-related work stress and counterproductive work behavior. Time pressure has been widely used in workplace research, and some studies have directly shown that perceived time pressure has a positive effect on knowledge hiding (Zhang et al., 2021). This has laid a good foundation for the research of this paper. In China, the new generation of employees have been placed on the stage with the changes of times. They work in the stressful environment. More and more employees work from nine in the morning to nine in the evening on Monday to Saturday. This study pay attention to this time pressure. The pressure seems to be a reason for negative behavior.

Further, territoriality, a concept developed on the basis of psychological ownership (Ma and Gao, 2010), has rarely been studied in the Chinese context. Territoriality originated from zoology. At a primary stage, it investigates the behavior of animals occupying territory to study the evolutionary traits of organisms. Additionally, it studies human territoriality and territorial behavior. Unlike animal territoriality, humans not only have biological evolutionary characteristics (Edney, 1974) but also understand territoriality as an individual’s sense of possession of his/her own things and a sense of preventing others from encroachment (Chu and Yang, 2011). In particular, previous studies have shown that territoriality is closely linked to knowledge hiding (Peng, 2013). Therefore, this study includes territoriality in its analysis.

This paper focuses on the factors influencing knowledge hiding behavior in the Chinese context. The sample was from companies in China. This studied also can be used in other areas of China and other countries. They are outstanding problems in China. The study on this context is representative. It has been verified that employees around the world all have knowledge hiding behavior. American Management Association’s studies in 2008 indicated that employees are generally reluctant to share their knowledge (Haas and Park, 2010). IDC’s studies shows that the global 500 companies lost 31.5 billion without effective knowledge sharing every year (Li and Huang, 2018). The same is true in China. 46% employees in China had hidden their knowledge (He and Jiang, 2014). Time pressure has been widely used in workplace research, and some studies have directly shown that perceived time pressure has a positive effect on knowledge hiding (Zhang et al., 2021). Time pressure also exists in other countries (De Clercq et al., 2019). The relation between knowledge hiding and territoriality had been verified abroad (Singh, 2019). So we believe that the analysis and discussion in this paper could cover these stressful phenomena. They can represent a part of same problem about knowledge hiding in other countries. Thus, this study investigates the influence mechanism of justice sensitivity on knowledge hiding by focusing on knowledge hiding in the process of knowledge communication of enterprise employees and introducing multiple variables such as perceived time pressure and territoriality.

## Literature Review and Hypotheses Development

### Justice Sensitivity and Knowledge Hiding

Based on (Schmitt et al., 2005) classification of unfair events, justice sensitivity can be divided into victim sensitivity, perpetrator sensitivity, and observer sensitivity. Further, perpetrator sensitivity can be divided into two categories perpetrator sensitivity and beneficiary sensitivity (Schmitt et al., 2010). This study only selects victim sensitivity and perpetrator sensitivity for two reasons. First, studies have shown that observer sensitivity and beneficiary sensitivity have a high correlation, with non-ideal discriminant validity (Xie et al., 2013). Second, since the sensitivities of victims and beneficiaries to unfair events under the circumstance of active participation can be regarded as two opposite types, it aims to compare and study the knowledge hiding of these two justice sensitivity types.

Victim sensitivity predicts peoples’ behaviors in a social dilemma. As people with high sensitivity might perceive themselves as victims of unfair situations such as destiny and are less likely to trust others, they usually show noncooperation, hostility, and even vindictiveness (Gollwitzer and Rothmund, 2011; Stavrova et al., 2014) because of the psychology of self-protection (Gerlach et al., 2012). According to the research on knowledge hiding, distrust is positively related to knowledge hiding (Kumar Jha and Varkkey, 2018) while a significant cause of knowledge hiding is self-protection (Peng, 2013). When knowledge is unique to individuals, it could provide a competitive advantage for people in organizations. Therefore, we propose the following hypothesis:

H1a: Victim sensitivity is positively related to knowledge hiding.

When being unfairly treated, perpetrators usually think they have violated the social or organizational justice rules (Stavrova et al., 2014) and thus have a sense of guilt and tend to make up for their own mistakes. Therefore, personal guilt has a negative impact on knowledge hiding (Fang, 2017). Moreover, the perpetrators’ sensitivity is positively correlated with humility and gentleness, and positively predicts prosocial tendencies, such as solidarity with vulnerable others (Baumert et al., 2014). Thus, perpetrators with high sensitivity tend to share more to gain more benefits, rather than asking others to share as losers (Stavrova and Schlösser, 2015). Hence, we postulate the following hypothesis:

H1b: Perpetrator sensitivity is negatively related to knowledge hiding.

### Justice Sensitivity and Perceived Time Pressure

Although victim sensitivity and perpetrator sensitivity elicit different inner activities and emotional tendencies, people with high sensitivity of both types are reflected in their sensitivity to injustice. In general, people with high justice sensitivity can perceive more information about injustice (Chi et al., 1981; Schneider and Bjorklund, 1992). In corporate work, when employees perceive time pressure, they think that individual time resources have been deprived, which is an unfair event, with people with high justice sensitivity being more likely to detect such unfair events (Baumert et al., 2011). According to the conservation of resources theory, individuals regard potential or actual resource loss as a threat (Hobfoll, 1989). Therefore, individuals with high victim sensitivity have a high degree of perception of their own adverse situations and psychological prevention construction. Although perpetrators with high sensitivity are the ones who gain benefits, studies show that perpetrators with high sensitivity also perceive more pressure and loss of resources (Chi et al., 1981). Hence, we put forward the following hypotheses:

H2a: Victim sensitivity is positively related to perceived time pressure.

H2b: Perpetrator sensitivity is positively related to perceived time pressure.

### Justice Sensitivity and Territoriality

Studies on the influence of perceived organizational justice on organizational citizenship behavior and psychological ownership have found that perceived organizational justice could increase organizational citizenship behavior and psychological ownership (Mohammad et al., 2019). Further, individual and organizational psychological ownerships are often in opposition. Whereas individual psychological ownership pays more attention to the individual, organizational ownership focuses on the organization. In addition, some studies have shown that employee psychological ownership has a positive impact on territoriality (Peng, 2013). Moreover, organizational justice refers to the employees’ perceptions of fairness in an organization, which increases organizational psychological ownership. This further indicates that organizational justice has a negative effect on employee psychological ownership, and consequently, territoriality because employee psychological ownership has a positive impact on territoriality.

From the victim’s viewpoint, the victim, as the aggrieved party, will be filled with feelings of injustice. In particular, organization injustice can increase the staff’s personal psychological ownership, while personal psychological ownership increases territoriality. Therefore, victim sensitivity has a positive effect on territoriality.

From the perspective of perpetrators, who obtain the benefits, their sense of the organization fairness will increase, and think that the organization itself allow them to obtain more benefits. Therefore, people with high perpetrator sensitivity will have higher organizational psychological ownership, that is, a reduction in personal psychological ownership, and consequently, a reduction in territoriality. Since the perpetrators tend to share more to gain more benefits (Chi et al., 1981), perpetrator sensitivity should have a negative impact on territoriality. Hence, we postulate the following hypotheses:

H3a: Victim sensitivity is positively related to territoriality.

H3b: Perpetrator sensitivity is negatively related to territoriality.

### Perceived Time Pressure and Territoriality

When individual resources are deprived, individuals tend to take priority actions to protect their own resources to avoid the continuous loss of resources (Cheek and Buss, 1981). According to the conservation of resources theory, time can be regarded as an individual resource. When animals feel time pressure, they will feel deprived of individual resources, thus evoking the will and actions to protect their personal resources, which will increase their territoriality. For example, in response to the increased intruder pressure at the time of dawn, a critical period for vocal displays, songbirds can increase the singing rates (Hill et al., 2017). Other studies from the field of zoology show that time pressure has an impact on territoriality (Wronski and Plath, 2006), The above conclusion is in turn extended to anthropological research, which shows that in the retail environment, the impending closing time will lead to the employees’ feeling of territoriality invasion, and consequently, the employees’ territorial behavior (Ashley and Noble, 2014). Thus, we put forward the following hypothesis:

H4: Perceived time pressure is positively related to territoriality.

### The Mediating Effect Between Perceived Time Pressure and Territoriality

In today’s competitive environment, enterprises and organizations of different sizes have their own performance evaluation systems and adopt different approaches to improve their performance including the use of their own unique advantages or personal tacit knowledge. Since knowledge is inherently exclusive and monopolistic, in a competitive environment, individuals need to ensure that their knowledge resources are not stolen by others in pursuit of higher performance, thus maintaining their advantage and showing a territoriality behavior. Simultaneously, in a competitive environment, people with high justice sensitivity will pursue fairer competition, as justice sensitivity affects their territoriality, and consequently, knowledge hiding (Peng, 2013). Therefore, territoriality may mediate the relationship between justice sensitivity and knowledge hiding.

However, with the prevalence of overtime work in today’s society, time deprivation is becoming a serious phenomenon, making people with high justice sensitivity more concerned about whether they will get adequate compensation for their deprived time. Therefore, they will be more sensitive to time deprivation, that is, they will perceive a greater time pressure, thus increasing knowledge hiding (Škerlavaj et al., 2018). Therefore, perceived time pressure may mediate the relationship between justice sensitivity and knowledge hiding.

According to the theory of conservation of resources, when employees feel pressure, they need to obtain new resources from the outside world to compensate for the lost resources, or they will take more strict actions to protect the resources they own (Wu et al., 2012). Victim sensitivity can improve people’s perceived pressure, with existing studies showing that perceived time pressure has an impact on knowledge hiding (Škerlavaj et al., 2018). Facing time pressure, people will discover more possibilities of territoriality invasion and engage in territorial behavior to influence other people. Moreover, existing studies show that territoriality can affect knowledge hiding (Peng, 2013). Based on hypotheses H1, H2, H3, and H4, we further propose that perpetrator sensitivity has an indirect effect on knowledge hiding. Although the indirect paths of perpetrator sensitivity and victim sensitivity are similar, the mechanisms differ under the influence of perceived time pressure and territoriality. The specific hypotheses are as follows:

H5a: Perceived time pressure mediates the relationship between victim sensitivity and knowledge hiding.

H5b: Perceived time pressure mediates the relationship between perpetrator sensitivity and knowledge hiding.

H6a: Territoriality mediates the relationship between victim sensitivity and knowledge hiding.

H6b: Territoriality mediates the relationship between perpetrator sensitivity and knowledge hiding.

H7a: Perceived time pressure and territoriality play a chain-mediating role between victim sensitivity and knowledge hiding.

H7b: Perceived time pressure and territoriality play a chain-mediating role between perpetrator sensitivity and knowledge hiding.

### The Moderating Effect of Territoriality

According to the theory of conservation of resources, time is a personal resource of people. Thus, when people feel time pressure, the resources are occupied. For people with high territoriality, they care more about their territory being violated, thus evoking a more reactive defensive behavior, namely, the more the resources of a person that are encroached, the higher the probability of a knowledge-hiding behavior. In addition, territoriality plays a moderating role between perceived time pressure and knowledge hiding, that is, the higher the degree of aggression against other people’s territory, the higher the sense of injustice, the greater the anger, and the more aggressive the reactive defensive behavior (Brown and Robinson, 2011). Therefore, people’s self-defense will increase when their resources are violated, and an increase in self-defense may lead to an increase in knowledge hiding. Through the influence of justice sensitivity on perceived time pressure, knowledge hiding is influenced by territoriality performance. Therefore, it can be inferred that territoriality has a moderating effect on the two mediating pathways of justice sensitivity and knowledge hiding. Thus, it is assumed in this study that the higher the territoriality, the more likely people are to engage in knowledge hiding behaviors under time pressure. Therefore, we propose the following hypotheses:

H9: Territoriality positively moderates the relationship between perceived time pressure and knowledge hiding.

H10a: Territoriality positivity moderates the relationship between victim sensitivity and knowledge hiding through perceived time pressure.

H10b: Territoriality positivity moderates the link between perpetrator sensitivity and knowledge hiding through perceived time pressure.

**FIGURE 1:**
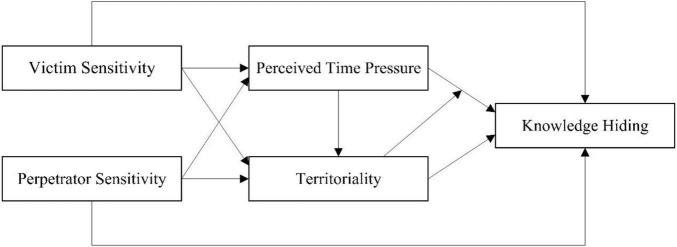
Theoretical model.

We take territoriality as both mediator variable and moderator variable. Firstly, it is feasible for a variable to act as both a mediator variable and a moderator variable (Ma, 2012). According to the meanings of territoriality, we take it as both mediator variable and moderator variable. First, the level of each individual territoriality is different (Brown et al., 2005). People react differently to feeling injustice and stress between high level and low level of territoriality. It also makes a difference in the part of knowledge hiding behavior. People’s territoriality is also disturbed by other factors. For example, territoriality may be a normal range in the general state. It changes if people are subjected to a particular stimulus such as justice sensitivity and perceived time pressure (Peng, 2011). Territoriality could be as the moderator variable. It is affected by justice sensitivity and perceived time pressure. Then, knowledge hiding behavior is affected by territoriality. Based on the above hypotheses, we construct the following theoretical model in Figure 1.

## Materials and Methods

### Measuring Tools

In this study, we used a total of five variables (i.e., victim sensitivity, perpetrator sensitivity, perceived time pressure, territoriality, and knowledge hiding). All the scales used in this study are mature and have been repeatedly studied and verified. After the pre-survey, the formal items were determined and scored on a 7-point Likert scale; the higher the score, the stronger the corresponding trend.

We adopted the scale developed by Schmitt (Schmitt et al., 2010), the author of the justice sensitivity theory, to measure victim sensitivity. The scale includes nine items, such as “I am very upset when others receive something that should belong to me.” For perpetrator sensitivity, we adopted another scale developed by Schmitt et al. (Schmitt et al., 2010), which includes 10 questions, such as “I feel depressed when I take something from others that I should not have.” The scale developed by Putrevu et al. was used to measure perceived time pressure. Following Škerlavaj et al. (2018), we verified that the scale includes three items, such as “When I need to complete a task, I find myself short of time.” For territoriality, we used a scale developed by Avey et al. (2009) and validated by Peng (2013) which includes 3 questions such as “I protect my ideas from being used by others in the organization.” For knowledge hiding, we used the scale developed by Connelly et al. (2012), the author of the concept, which includes 12 items, such as “Pretend I don’t know relevant information when colleagues ask me about knowledge”.

Moreover, according to the results of previous studies, some demographic variables were selected as control variables, such as including gender, age, working years, education, enterprise nature, industry, and position level.

The paper design the scale questions are from foreign mature scale. It applies to this article research object. The article’s research object includes perpetrator sensitivity, victim sensitivity, territoriality, perceived time pressure and knowledge hiding. Their meaning is similar under different backgrounds of culture. These scales have been used in Chinese literature. They had been verified that they are validated in the Chinese context (Peng, 2011; Liu et al., 2019; Zhang et al., 2021). Hence, we choose these fully fledged scales.

### Sample Characteristics

The questionnaires for this study were collected from China through the Wenjuanxing Sample Service. A total of 436 questionnaires were collected, of which 391 were valid, with a recovery rate of 90.0%. The descriptive statistical results are shown in Table 1. (It is the content about descriptive statistics in Appendix A).

**TABLE 1:** Distribution of demographic characteristics of samples.

Characteristic	Category	Proportion(%)	Characteristic	Category	Proportion(%)
Gender	Male	44.8	Enterprise nature	state-owned enterprise	23.5
	Female	55.2		private enterprise	55.0
Age	< 18	0		jointly operated enterprise	15.6
	18–25	24.8		Others	5.9
	26–39	73.9	Industry	manufacturing	31.2
	> 40	1.3		Construction	9.7
Work experience	< 1	2.0		Finance	13.0
	1–3	24.6		information technology service industry	28.4
	3–5	24.6		wholesale and retail	5.9
	5–10	39.1		Others	11.8
	> 10	9.7	Position level	ordinary frontline staff	31.5
Education	senior high school and below	2.3		frontline managers	38.9
	junior college	11.0		middle managers	26.9
	Undergraduate	74.2		top management	2.8
	Master’s degree and higher	12.5			

### Data Verification

The reliability and validity of the test results are presented in Table 2. The Cronbach’s alpha values of the variables in this study were all greater than 0.7, indicating good reliability, the composite reliability (CR) values of all variables were greater than 0.7, and the average variance extracted (AVE) values were greater than 0.5. The shaded part in the table is the square root of AVE, which was greater than the correlation coefficient between the corresponding variable and other variables, indicating good discriminative validity. The degree of variation of the cumulative explanatory variance of the five common factors was 62.36%, among which the variation degree of the explanatory variance of the first factor was 26.60%, but did not exceed 40%, indicating that there was no common method deviation (Zhao and Xu, 2020). The data analysis was performed using the SPSS software.

**TABLE 2:** Reliability, validity, and correlation coefficients of latent variables.

	Cronbach’s Alpha	CR	AVE	Knowledge Hiding	Victim Sensitivity	Perpetrator Sensitivity	Perceived Time Pressure	Territoriality
Knowledge Hiding	0.949	0.951	0.623	0.789				
Victim Sensitivity	0.900	0.902	0.506	0.251**	0.711			
Perpetrator Sensitivity	0.899	0.912	0.510	0.088	0.303**	0.714		
Perceived Time Pressure	0.829	0.835	0.628	0.295**	0.379**	0.312**	0.792	
Territoriality	0.840	0.788	0.553	0.279**	0.368**	0.173**	0.348**	0.743

**p < 0.01. The square roots of the AVEs are the bottom shadow numbers on the diagonal line.

## Results

### Multiple Regression Analysis

Hierarchical regression was conducted, and the results are presented in Table 3. They show that victim sensitivity has a significant positive predictive effect on knowledge hiding (a standardization coefficient of 0.218, *P* < 0.001), thus verifying H1a. However, perpetrator sensitivity has no significant negative predictive effect on knowledge hiding (a standardization coefficient of 0.015, *P* > 0.05) thus rejecting H1b. After including victim sensitivity, perpetrator sensitivity, territoriality, and perceived time pressure in the regression equation, the predictive effects of territoriality and perceived time pressure on knowledge hiding were found to be significant at the 0.01 level, whereas perpetrator sensitivity was still not significant. In addition, victim sensitivity has a significant positive effect on perceived time pressure (a standardization coefficient of 0.315, *P* < 0.001) and perpetrator sensitivity has a significant effect on perceived time pressure (a standardization coefficient of 0.215, *P* < 0.001) thus supporting H2A and H2B. The effects of victim sensitivity and perceived time pressure on territoriality were found to be both significant at the 0.001 level, whereas perpetrator sensitivity was not significant, thus supporting H3A and H4 and rejecting H3B.

**TABLE 3:** Regression analysis results.

Variable	Knowledge Hiding		Territoriality	Perceived Time Pressure
Gender	–0.028	–0.040	0.020	0.041
Age	–0.118	−0.123*	0.020	0.006
Work experience	–0.102	–0.089	–0.050	–0.021
Education	–0.072	–0.049	−0.104*	–0.021
Enterprise nature 1	0.085	0.063	0.106	0.021
Enterprise nature 2	0.021	0.024	0.057	–0.054
Enterprise nature 3	0.042	0.041	0.078	–0.054
Industry 1	–0.050	–0.037	–0.041	–0.025
Industry 2	0.016	0.019	–0.003	–0.011
Industry 3	0.131	0.125	–0.054	0.069
Industry 4	–0.016	0.009	–0.050	–0.075
Industry 5	–0.002	0.001	–0.055	0.025
Position level	0.156**	0.150**	–0.044	0.062
Victim Sensitivity	0.218***	0.103	0.266***	0.315***
Perpetrator Sensitivity	0.015	–0.037	0.028	0.215***
Perceived Time Pressure		0.182**	0.240***	
Territoriality		0.167**		
R^2^	0.130	0.191	0.210	0.210
F	3.737***	5.180***	6.207***	6.637***

*p < 0.05; **p < 0.01; ***p < 0.001.

### Mediating Effect Analysis

#### ① The Mediating Effect of Victim Sensitivity on Knowledge Hiding

In this study, the bootstrap method (5000 samples) was used to investigate the chain mediating effect of perceived time pressure and territoriality between victim sensitivity and knowledge hiding using Process3.3 plugin. Gender, age, working years, educational level, industry, enterprise nature, position level, and perpetrator sensitivity were used as control variables. The results of the mediating effect analysis are presented in Table 4. The results show that the direct effect of victim sensitivity on knowledge hiding is not significant. The bootstrap 95% confidence interval included zero; thus, the perceived time pressure and territoriality play a complete mediating role between victim sensitivity and knowledge hiding. The total effect was 0.218, and the confidence interval did not contain zero, indicating significance. The confidence intervals of the three mediating effects did not contain zero, indicating that all are significant. The total mediating effect value was 0.115, accounting for 52.8% of the total effect. The path of indirect effect 1 was victim sensitivity → perceived time pressure → knowledge hiding, with an effect value of 0.057, accounting for 26.3% of the total effect ratio. The path of indirect effect 2 was victim sensitivity → territoriality → knowledge hiding, with an effect value of 0.045, accounting for 20.4% of the total effect ratio. The path of indirect effect 3 was victim sensitivity → perceived time pressure → territoriality → knowledge hiding. The results show that the chain mediating effect was significant, accounting for 6.20% of the total effect. Further, 1-3 is the difference between indirect effect 1 and indirect effect 3, and the result was significant, as is 2–3, thus supporting H5A, H6A, and H7A.

**TABLE 4:** The mediating effect of victim sensitivity on knowledge hiding.

	Effect	BootSE	BootLLCI	BootULCI	Percentage to total effect
Total effect	0.218	0.053	0.114	0.322	100%
Direct effect	0.103	0.055	–0.006	0.212	–
Total indirect effect	0.115	0.028	0.065	0.173	52.8%
Indirect effect 1	0.057	0.020	0.023	0.102	26.1%
Indirect effect 2	0.045	0.018	0.014	0.085	20.6%
Indirect effect 3	0.013	0.005	0.004	0.024	6.0%
1 minus 2	0.013	0.029	–0.042	0.070	–
1 minus 3	0.045	0.020	0.010	0.087	20.6%
2 minus-3	0.032	0.017	0.004	0.071	14.7%

#### ② The Mediating Effect of Perpetrator Sensitivity on Knowledge Hiding

The mediating effect of perceived time pressure and territoriality on perpetrator sensitivity and knowledge hiding are shown in Table 5. The results show that the direct effect of perpetrator sensitivity on knowledge hiding was not significant. The bootstrap 95% confidence interval contained zero; thus, the perceived time pressure and territoriality play a complete mediating role between perpetrator sensitivity and knowledge hiding, with a total effect of 0.089 (the absolute value of indirect effect + direct effect). The total indirect effect value was 0.052, accounting for 58.4% of the total effect. The path of indirect effect 1 was perpetrator sensitivity → perceived time pressure → knowledge hiding, with an effect value of 0.039, accounting for 43.8% of the total effect ratio. The confidence interval did not contain zero, indicating significance. The path of indirect effect 2 was perpetrator sensitivity → territoriality → knowledge hiding, with an effect value of 0.005, and a confidence interval that contained 0; thus, not significant. The path of indirect effect 3 was perpetrator sensitivity → perceived time pressure → territoriality → knowledge hiding. This chain mediation effect was found to be significant, accounting for 10.1% of the total effect. 1-2 is the difference between indirect effect 1 and indirect effect 2, and the result was significant, as is 1-3, thus supporting, H5B and H7B and rejecting H6B.

**TABLE 5:** The mediating effect of perpetrator sensitivity on knowledge hiding.

	Effect	BootSE	BootLLCI	BootULCI	Percentage to total effect
Total effect	0.015	0.052	–0.088	0.118	–
Direct effect	–0.037	0.052	–0.139	0.065	–
Total indirect effect	0.052	0.018	0.019	0.090	58.4%
Indirect effect 1	0.039	0.014	0.015	0.068	43.8%
Indirect effect 2	0.005	0.011	–0.017	0.027	–
Indirect effect 3	0.009	0.004	0.002	0.019	10.1%
1 minus 2	0.034	0.018	0.001	0.072	38.2%
1 minus 3	0.030	0.013	0.007	0.059	33.7%
2 minus-3	–0.004	0.012	–0.032	0.019	–

### Moderating Effect Analysis

#### ① The Moderating Role of Territoriality Between Perceived Time Pressure and Knowledge Hiding

Using Process3.3, victim sensitivity and perpetrator sensitivity and demographic variables were used as control variables. The results are listed in Table 6. Int_1 is the perceived time pressure × territoriality, with significant interaction term, indicating that the moderating effect is significant.

**TABLE 6:** Regression coefficients and significance.

	Knowledge Hiding			
	Effect	BootSE	BootLLCI	BootULCI
Perceived Time Pressure	0.184	0.054	0.079	0.290
Territoriality	0.190	0.053	0.085	0.295
Int_1	0.083	0.041	0.003	0.163

**FIGURE 2:**
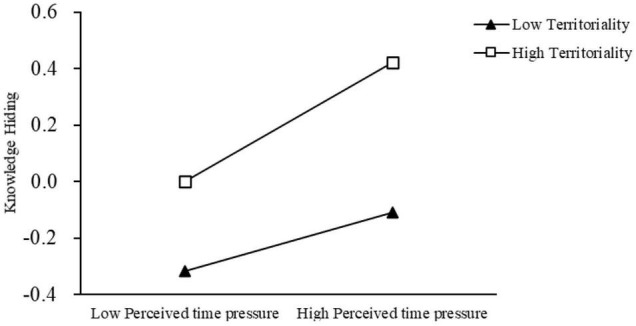
Moderating effects of different perceived time pressure levels.

Figure 2 shows a moderating effect diagram. The results show that, regardless of the level of perceived time pressure, territoriality plays a positive moderating role between perceived time pressure and knowledge hiding, and the effect of perceived time pressure on knowledge hiding is always positive, thus supporting H9.

#### ② The Moderating Role of Territoriality Between Victim Sensitivity and Knowledge Hiding

We tested the moderating effect of the territoriality in the mediating path from victim sensitivity to knowledge hiding. The results are shown in Table 7, where Int_1 is the perceived time pressure × territoriality. In this mediation model, the moderating effect of territoriality was significant.

**TABLE 7:** Moderated mediating regression coefficients and significance of victim sensitivity.

	Knowledge Hiding			
	Effect	BootSE	BootLLCI	BootULCI
Victim Sensitivity	0.108	0.055	−0.009	0.216
Perceived Time Pressure	0.184	0.054	0.079	0.290
Territoriality	0.190	0.053	0.085	0.295
Int_1	0.083	0.041	0.003	0.163

Table 8 shows the results of the mediating effect analysis. The results show that the mediating effect was 0.032 and the confidence interval contained 0 when subtracting one standard deviation from the moderator variable territoriality, indicating an insignificant mediating effect. When one standard deviation was added, the mediating effect increased to 0.084 with a confidence interval that did not contain zero, indicating a significant mediating effect. It can be seen that the mediating effect has significant changes under the regulation of territoriality. Further, the confidence interval of Index did not contain zero (Hayes, 2015), thus further indicating that the mediation model is valid. In the table, 2-1 represents the difference between the mediating effect under M and M-1SD, 3-1 represents the difference between M+1SD and M-1SD, and 3-2 represents the difference between M+1SD and M. The results were all significant, thus supporting H10a and confirming the validity of the mediation model.

**TABLE 8:** Moderated mediating effect of victim sensitivity.

	Moderated mediating effect			
	Effect	BootSE	BootLLCI	BootULCI
M-1SD	0.032	0.020	−0.001	0.076
M	0.058	0.019	0.025	0.100
M+1SD	0.084	0.027	0.037	0.143
Index	0.026	0.013	0.001	0.053
2 minus 1	0.026	0.013	0.001	0.053
3 minus 1	0.053	0.027	0.002	0.106
3 minus 2	0.026	0.013	0.001	0.053

**FIGURE 3:**
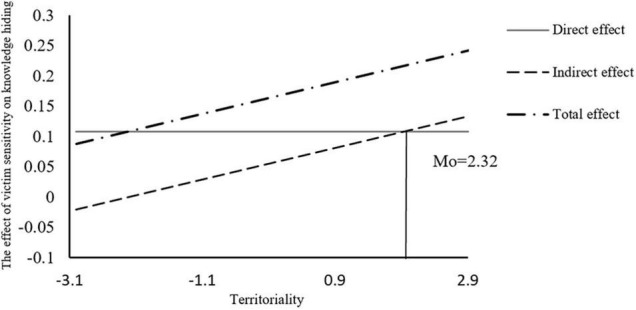
Slope diagram of the mediating effect of victim sensitivity.

As shown in Figure 3, for the mediating effect of slope variation, as this can regulate the mediating effect during the second half only, and the direct effect of victim sensitivity on knowledge hiding was not significant, the direct effect is represented as a straight line parallel to the x-axis. With the augmentation of the regulating effect, the indirect effect becomes increasingly large, and simultaneously the total effect also increases. When Mo = 2.32, the direct and indirect effects are equal, then the indirect effect becomes greater than the direct effect.

**FIGURE 4:**
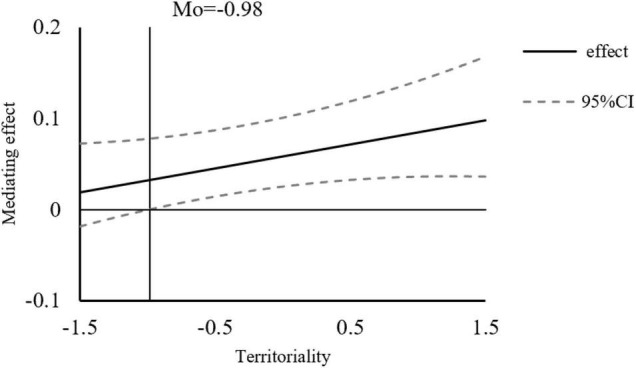
Moderated mediating effect and confidence interval of victim sensitivity.

According to the results in Table 8, in the case of a negative one standard deviation, the indirect effect of victim sensitivity on knowledge hiding was not significant. In this study, we use continuous control variables. Additionally, the collocation method (Preacher et al., 2007) is used to estimate the mediation effect of the simple slope and to investigate the mediation effect significant points. Specific results are shown in Figure 4. When the territoriality is greater than −0.98, the mediating effect is significant, and with the increase of the moderating effect, the mediating effect becomes stronger, that is, the territoriality has a positive moderating influence on the relationship between victim sensitivity and knowledge hiding.

#### ③ The Moderating Role of Territoriality Between Perpetrator Sensitivity and Knowledge Hiding

The moderating effect of territoriality in the mediating path from perpetrator sensitivity to knowledge hiding was tested. The results are shown in Table 9, where Int_1 is the perceived time pressure × territoriality. In this mediating model, the moderating effect of territoriality was significant.

**TABLE 9:** Moderated mediating regression coefficients and significance of perpetrator sensitivity.

	Knowledge Hiding			
	Effect	BootSE	BootLLCI	BootULCI
Perpetrator Sensitivity	−0.028	0.052	−0.129	0.074
Perceived Time Pressure	0.184	0.054	0.079	0.290
Territoriality	0.190	0.053	0.085	0.295
Int_1	0.083	0.041	0.003	0.163

The moderated mediating effect analysis is presented in Table 10. The results show that the mediating effect was 0.022 and the confidence interval contained zero when subtracting one standard deviation from the moderator variable of territoriality, and the mediating effect was not significant. When a standard deviation was added, the mediating effect increased to 0.057, the confidence interval did not contain zero, and the mediating effect was significant. It can be seen that the mediating effect changes significantly under the mediation of territoriality. Further, the confidence interval of Index did not contain zero, further indicating that the mediation model is valid. Comparisons between the mediating effects were also significant, thus supporting H10b and confirming the validity of the mediation model.

**TABLE 10:** Moderated mediating effect of perpetrator sensitivity.

	Moderated mediating effect			
	Effect	BootSE	BootLLCI	BootULCI
M-1SD	0.022	0.013	−0.001	0.050
M	0.040	0.014	0.016	0.070
M+1SD	0.057	0.020	0.023	0.102
Index	0.018	0.010	0.001	0.040
2 minus 1	0.018	0.010	0.001	0.040
3 minus 1	0.036	0.020	0.002	0.081
3 minus 2	0.018	0.010	0.001	0.040

**FIGURE 5:**
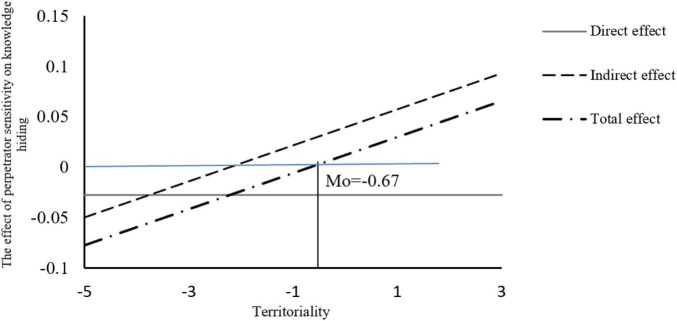
Slope diagram of the mediating effect of perpetrator sensitivity.

As shown in Figure 5, for the intermediary effect of slope variation, as this can regulate the mediation effect only during the second half, and the direct effect of perpetrator sensitivity on knowledge hiding was not significant, the direct effect is represented as a straight line parallel to the x-axis. With the augmentation of the regulating effect, the indirect effect becomes increasingly large, and simultaneously the total effect also increases due to the direct effect being negative. Therefore, when the total effect is equal to zero, the direct and indirect effects are equal. When Mo = 0.67, the direct and indirect effects are equal, then the indirect effect becomes greater than the direct effect.

**FIGURE 6:**
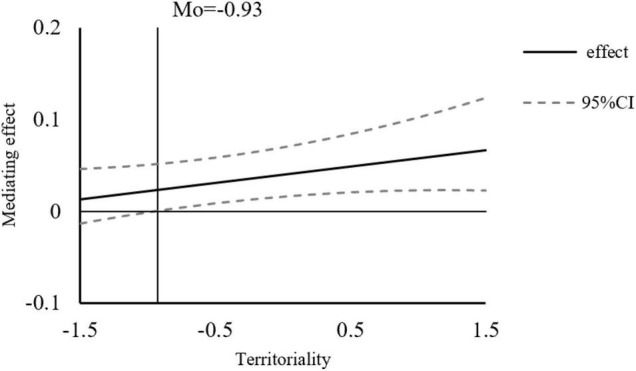
Moderated mediating effect and confidence interval of perpetrator sensitivity.

According to the results in Table 10, in the case of a negative one standard deviation, the indirect effect of perpetrator sensitivity on knowledge hiding was not significant. In this study, we use continuous control variables. Additionally, the collocation method is used to estimate the mediation effect of the simple slope and investigate the mediation effect significant points. Specific results are shown in Figure 6. When the territoriality is greater than −0.93, the mediating effect is significant, and with the increase of the moderating effect, the mediating effect becomes stronger, that is, the territoriality has a positive moderating effect on the relationship between perpetrator sensitivity and knowledge hiding.

## Discussion and Conclusion

### Main Conclusion

Based on the obtained results, we draw the following conclusions:

① Victim sensitivity has a positive direct effect on knowledge hiding, whereas perpetrator sensitivity has insignificant negative effect. In addition, the two justice sensitivities have significant impact on the perceived time pressure. It can be concluded that, regardless of the type, for people with high justice sensitivity, their sense of injustice around them will be enhanced, thus having a positive impact on perceived time pressure. Moreover, victim sensitivity has a positive effect on territoriality, indicating that victims tend to protect their own interests, thus increasing people’s territoriality, whereas the influence of perpetrator sensitivity on territoriality is not supported. The effect of perceived time pressure on territoriality is verified, which indicates that employees under time pressure will improve their territoriality behavior to protect their interests. On the one hand, perpetrator feel guilty. It will lead to reduce the knowledge hiding behavior (Fang, 2017). On the other hand, people with high perpetrator sensitivity are also highly sensitive to the injustice, which may reduce harmful behaviors. But it does not mean that will harm their own interests (Stavrova and Schlösser, 2015). The effect of perpetrator sensitivity on knowledge hiding is not significant because of the superposition of two kinds of psychological.

② Perceived time pressure can act as an independent mediating variable and play a mediating role. In particular, victim sensitivity can improve the perception of time pressure. Under time pressure, employees think that time resources have been occupied; thus, they will seek to make up for their loss of resources by engaging in knowledge hiding behaviors. The indirect path of territoriality as an independent mediating variable is also significant, indicating that people with high victim sensitivity will enhance their territoriality behavior to protect their interests from being damaged, while people with high territoriality will value their resource advantages, which will further promote the knowledge hiding behaviors. Since perception of time pressure improves people’s territoriality, victim sensitivity can cause knowledge hiding by increasing people’s perception of time pressure. In addition, although the total effect of perpetrator sensitivity on knowledge hiding is not significant, it is actually the result of the direct relationship between perpetrator sensitivity and knowledge hiding as well as the mediating effect of perceived time pressure and territoriality. Moreover, perpetrator sensitivity increases perceived time pressure, which in turn increases knowledge hiding behaviors. Therefore, the superposition of the positive indirect and negative direct effects (although not significant) leads to an insignificant relationship between perpetrator sensitivity and knowledge hiding.

③ The moderating effect of territoriality in both mediating paths is supported. The two types of justice sensitivities have a positive influence on the employees’ perception of time pressure, resulting in knowledge hiding behaviors through two indirect paths. People with high territoriality under time pressure will increasingly adopt a knowledge hiding behavior, whereas those with low territoriality, even under time pressure, are unlikely to hide knowledge. By adjusting the relationship between perceived time pressure and knowledge hiding, territoriality can further regulate the sensitivity of victims and perpetrators to knowledge hiding generated by perceived time pressure. The higher the territoriality, the higher the degree of justice sensitivity indirectly affecting knowledge hiding; whereas the lower the territoriality, the lower the degree of the indirect influence of justice sensitivity on knowledge hiding or the more insignificant the relationship.

### Theoretical Contributions

#### ① Identifying the Influencing Factors of Knowledge Hiding

Currently, there are few studies on the influence of individual factors on knowledge hiding despite its great significance to the literature on knowledge hiding at the individual level. In the past, many studies have shown that justice sensitivity has a significant effect on the psychology and behavior of employees; however, research in exploring the knowledge management field has been relatively insufficient. From the perspective of organizational justice, this paper investigates the mechanisms of employees’ knowledge hiding behavior to facilitate research and theory development.

#### ② Investigating the Influence of Justice Sensitivity on Outcome Variables

Since knowledge hiding is a type of self-protection behavior, and the theory of justice sensitivity can explain people’s self-protection behavior, this study focuses on justice sensitivity, studies its influence on knowledge hiding, and discusses the mechanisms of victim sensitivity and perpetrator sensitivity. Although some previous studies have combined the theories of resource protection and psychological ownership to explore the phenomenon of knowledge hiding, the relationship between justice sensitivity and knowledge hiding has not been sufficiently explored. Therefore, this study not only enriches the justice sensitivity theory and its relationship with knowledge hiding, but also explores its relationship with perceived time pressure and territoriality.

#### ③ Further Study on the Effect of Perceived Time Pressure and Territoriality on Knowledge Hiding

Time is an important resource, and under time pressure, people will think that their rights have been violated. Thus, it is meaningful to introduce the variable of perceived time pressure to explore the mechanism of people’s knowledge hiding. Moreover, according to the theory of psychological ownership of ideas, information, knowledge, and professional knowledge, the gain that people obtain when they invest considerable amounts of time and energy promotes all organizations to engage in territorial behaviors. Under this psychological effect, the impact of justice sensitivity on knowledge hiding behavior is mediated by the perceived time pressure and territoriality behavior. This implication is of great significance to the application of psychological ownership theory and the study of knowledge hiding and justice sensitivity. This study further explores the moderating effect of territoriality on the path of perceived time pressure as a single mediating variable.

### Practical Significance

This study has important practical implications and can provide new insights for the knowledge management of enterprises and organizations. In the case of injustice, the employee is either the victim or the perpetrator as the first participant. Victims are on the losing side of the injustice, and if they are not consoled at the organizational level, that will only increase their anger and lead to a more negative behavior. However, if the organization recognizes the existence of injustice, it should make timely compensations to the victims for their losses and provide favorable psychological conditions to protect the victims’ interests. Nevertheless, compensating the victims does not mean punishing the perpetrators. Although the perpetrators may cause knowledge hiding behaviors through various other factors, they are also in an unfair event. Even if the perpetrator is not the party whose interests are damaged, he/she feels that there is injustice in the organization and will continue to maintain his own interests in different ways. Therefore, organizations should provide perpetrators with a certain degree of psychological counseling to alleviate their inner feelings of guilt and alter their negative behavior. In order to mollify the sense of injustice and quench the anger and guilt, the management should take the effective organizational remedy (Zhu and Kou, 2014). The organization should lay down rationalized regulations to ensure justice. It could effectively protect the interests of employees and reduce knowledge hiding behavior.

In terms of working hours, although Chinese enterprises have a perfect overtime system and offer high salaries, if the time pressure reaches a certain level, employees may think that their loss of resources cannot be compensated by the salaries paid by the company, which will lead to the outbreak of a series of negative events. A number Therefore, enterprises should not blindly pursue results by making employees work excessively overtime. Instead, enterprises should have a certain degree of control, offer rationales for overtime work, and give high wages to the employees to compensate for their time, thus making the employees feel the worthiness of their overtime work and reducing the probability of engaging in knowledge hiding behaviors.

### Research Limitations and Future Prospects

First, this study discusses the regulating effect of territoriality, which acts as both a mediating and a moderating variable. However, in reality, there may be more external variables that regulate the model relationships. Second, limited to the theoretical model, the study fails to further explore the specific relationship between perpetrator sensitivity and territoriality. Thus, from the perspective of organizational justice, future studies can investigate the influence of other factors in the field of organizational justice and knowledge concealment, such as fairness sensitivity and other types of organizational justice. Thirdly, this paper did not extensively investigate the organizational knowledge systems. Therefore, future research can focus on knowledge in the organizational context with respect to the aspects of creation, innovation, and performance, and investigate the pre-variables and determinants that affect the knowledge hiding phenomenon. At last, justice sensitivity may be influenced by cultural characteristics. Thus, whether the relationship between justice sensitivity and other variables in this study is applicable to foreign cultural backgrounds needs further verification.

## Data Availability Statement

The original contributions presented in the study are included in the article/supplementary material, further inquiries can be directed to the corresponding author/s.

## Author Contributions

ZJ-S and JY-N designed the study. HH and RD-Y collected data and performed the data analysis. ZJ-S and JY-N checked the result. HH created figures. RD-Y made tables. ZJ-S and JY-N wrote, reviewed, and edited the manuscript. All authors wrote the manuscript and read and agreed to the published version of the manuscript.

## Conflict of Interest

The authors declare that the research was conducted in the absence of any commercial or financial relationships that could be construed as a potential conflict of interest.

## Publisher’s Note

All claims expressed in this article are solely those of the authors and do not necessarily represent those of their affiliated organizations, or those of the publisher, the editors and the reviewers. Any product that may be evaluated in this article, or claim that may be made by its manufacturer, is not guaranteed or endorsed by the publisher.

## Appendix A

### Here Are Descriptive Statistics

**TABLE A1:**

	Skewness					Kurtosis		
		Statistic		Standard Error		Statistic		Standard Error
Knowledge hiding1		0.846		0.123		−0.133		0.246
Perpetrator Sensitivity1		−1.071		0.123		1.553		0.246
Victim Sensitivity1		−1.251		0.123		1.828		0.246
Victim Sensitivity2		−0.841		0.123		0.351		0.246
Victim Sensitivity3		−0.654		0.123		−0.188		0.246
Victim Sensitivity4								

**TABLE A2:**

	*N*		Minimum	Maximum	Mean		Standard Deviation	Variance
	Statistic		Statistic	Statistic	Statistic		Statistic	Statistic
Knowledge hiding1	391		1.00	6.50	3.0488		1.34554	1.810
Perpetrator Sensitivity1	391		1.40	7.00	5.0972		1.00818	1.016
Victim Sensitivity1	391		1.22	6.78	4.9255		1.04523	1.093
Territoriality1	391		1.00	7.00	4.9838		1.29695	1.682
Perceived Time Pressure1	391		1.00	7.00	4.8261		1.23915	1.535
Valid *N* (List Status)	391							
